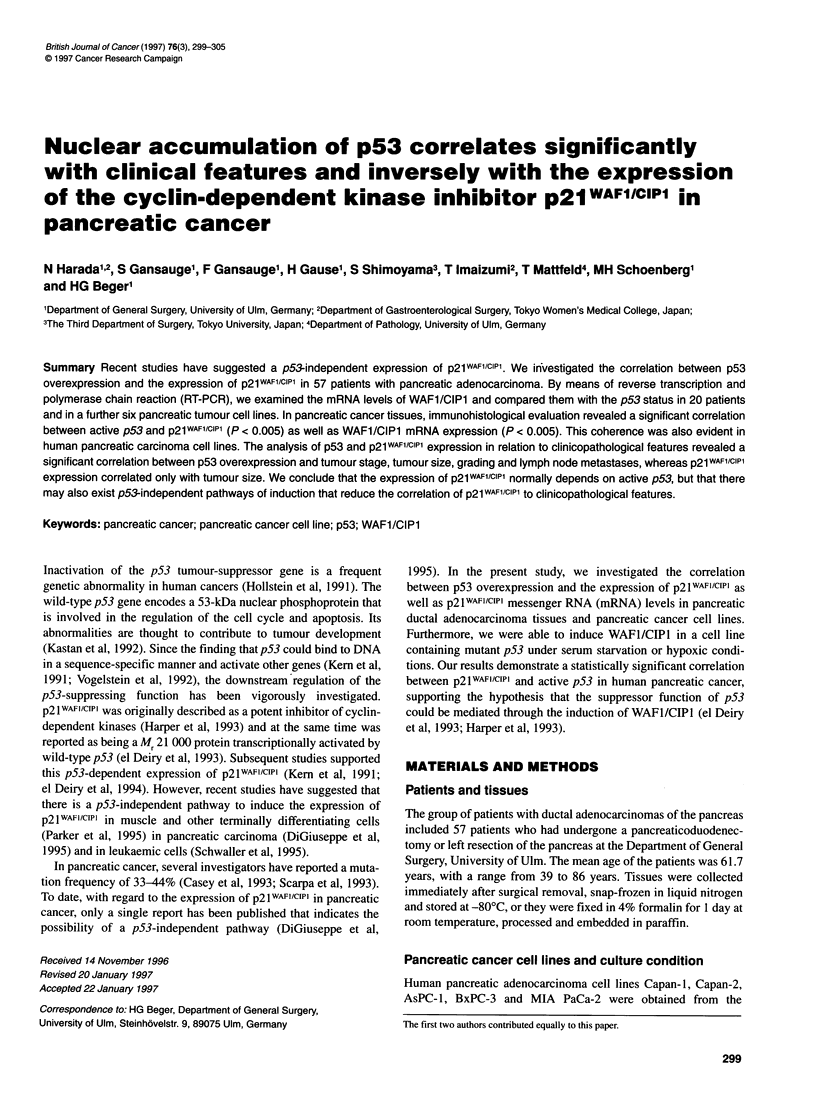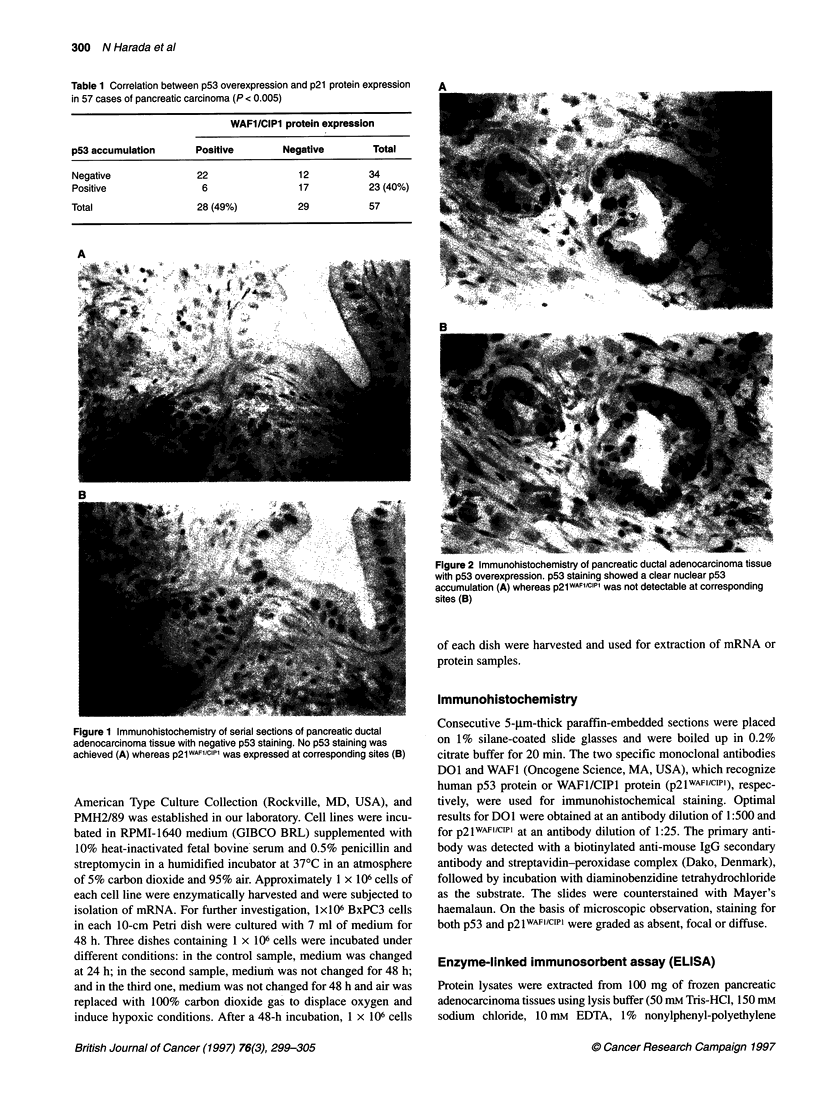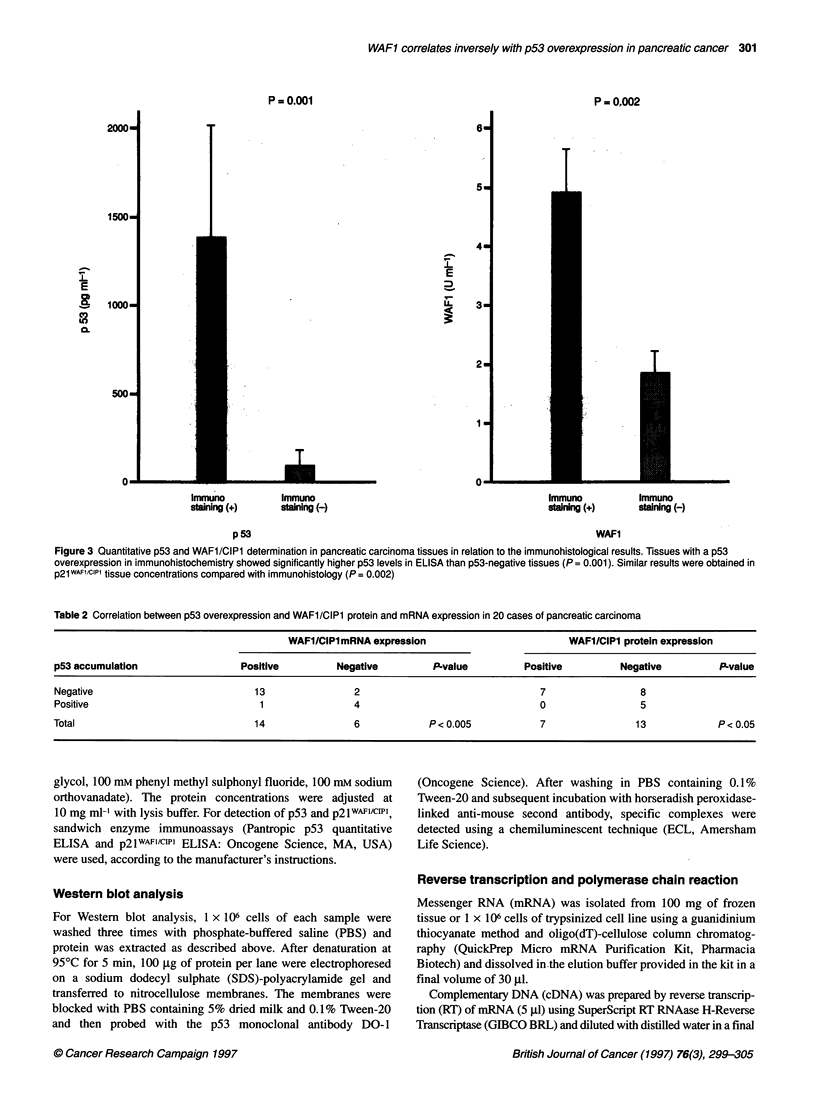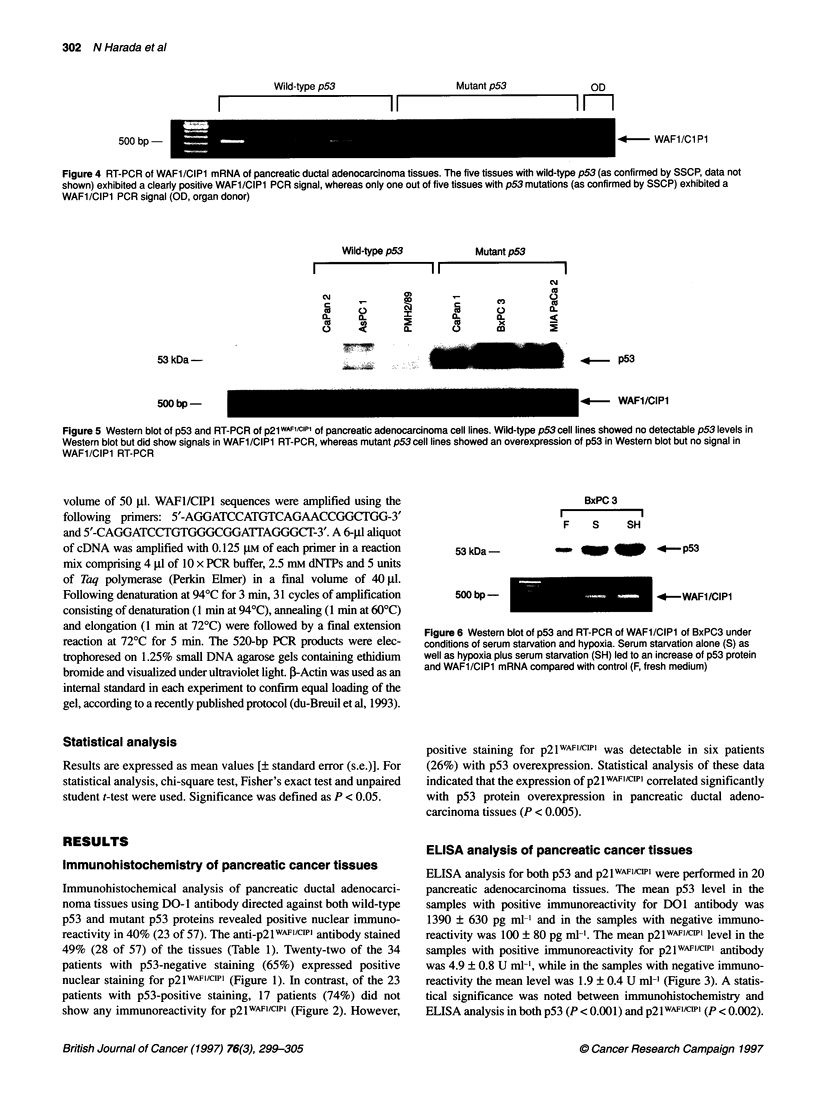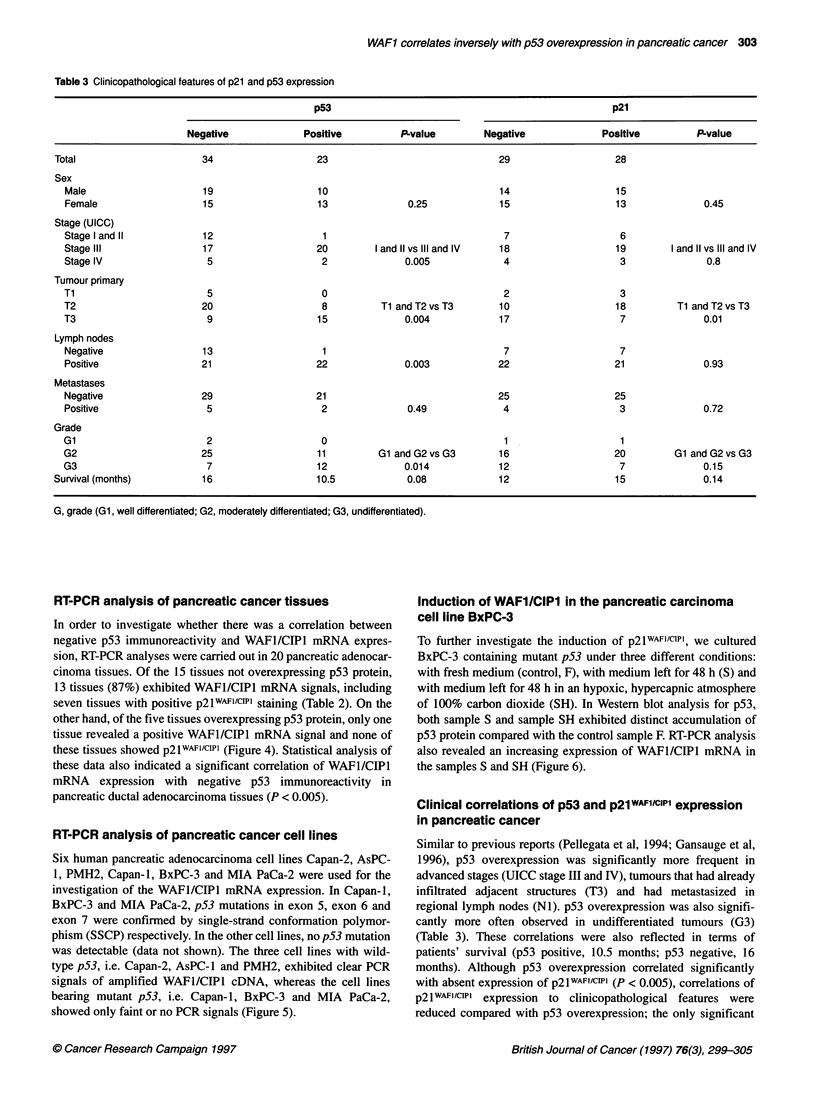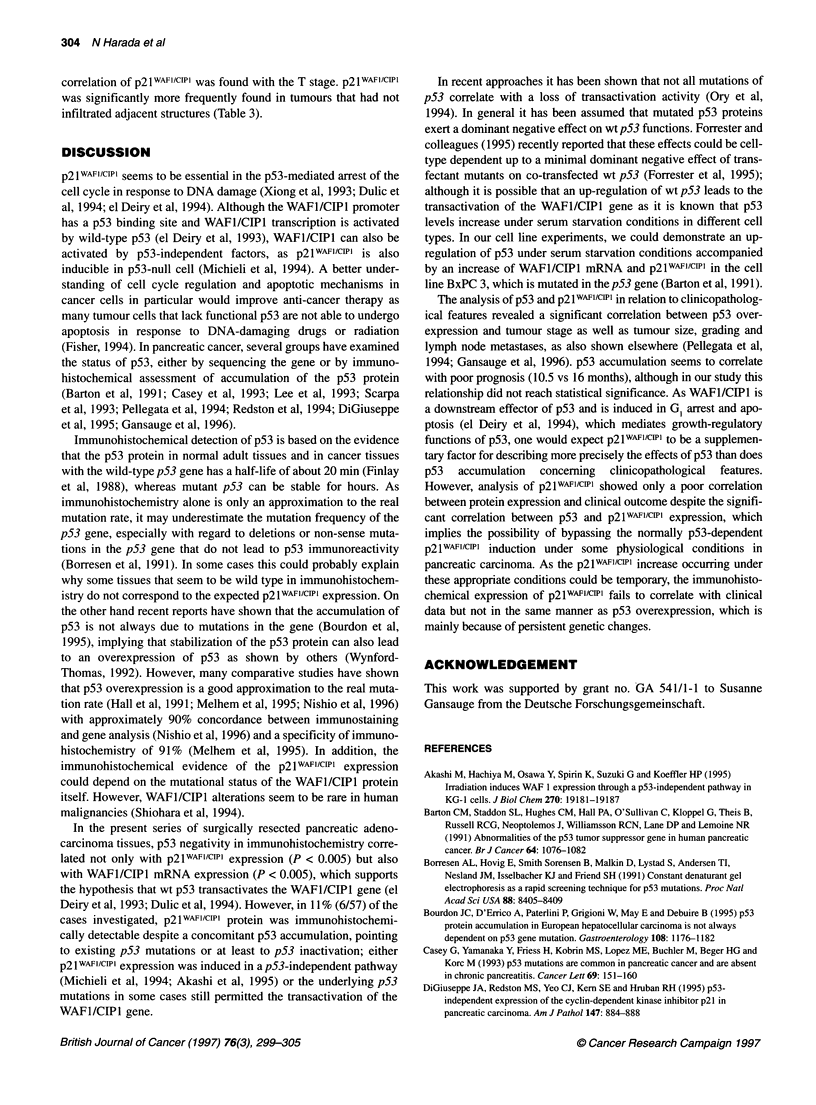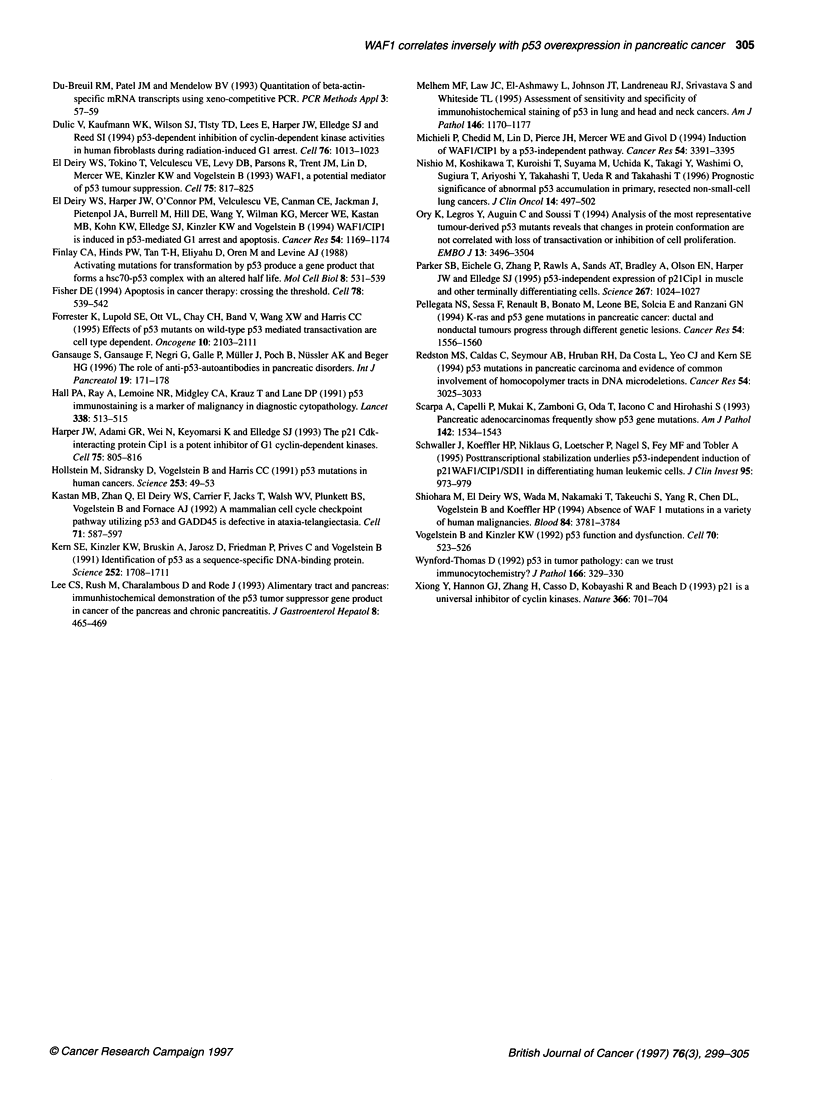# Nuclear accumulation of p53 correlates significantly with clinical features and inversely with the expression of the cyclin-dependent kinase inhibitor p21(WAF1/CIP1) in pancreatic cancer.

**DOI:** 10.1038/bjc.1997.382

**Published:** 1997

**Authors:** N. Harada, S. Gansauge, F. Gansauge, H. Gause, S. Shimoyama, T. Imaizumi, T. Mattfeld, M. H. Schoenberg, H. G. Beger

**Affiliations:** Department of General Surgery, University of Ulm, Germany.

## Abstract

**Images:**


British Journal of Cancer (1997) 76(3), 299–305
© 1997 Cancer Research Campaign

### Nuclear accumulation of p53 correlates significantly with clinical features and inversely with the expression of the cyclin-dependent kinase inhibitor p21$^{WAF1/CIP1}$ in pancreatic cancer

N Harada[1,2], S Gansauge[1], F Gansauge[1], H Gause[1], S Shimoyama[3], T Imaizumi[2], T Mattfeld[4], MH Schoenberg[1] and HG Beger[1]

[1]Department of General Surgery, University of Ulm, Germany; [2]Department of Gastroenterological Surgery, Tokyo Women's Medical College, Japan; [3]The Third Department of Surgery, Tokyo University, Japan; [4]Department of Pathology, University of Ulm, Germany

**Summary** Recent studies have suggested a *p53*-independent expression of p21$^{WAF1/CIP1}$. We investigated the correlation between p53 overexpression and the expression of p21$^{WAF1/CIP1}$ in 57 patients with pancreatic adenocarcinoma. By means of reverse transcription and polymerase chain reaction (RT-PCR), we examined the mRNA levels of WAF1/CIP1 and compared them with the *p53* status in 20 patients and in a further six pancreatic tumour cell lines. In pancreatic cancer tissues, immunohistological evaluation revealed a significant correlation between active *p53* and p21$^{WAF1/CIP1}$ ($P < 0.005$) as well as WAF1/CIP1 mRNA expression ($P < 0.005$). This coherence was also evident in human pancreatic carcinoma cell lines. The analysis of p53 and p21$^{WAF1/CIP1}$ expression in relation to clinicopathological features revealed a significant correlation between p53 overexpression and tumour stage, tumour size, grading and lymph node metastases, whereas p21$^{WAF1/CIP1}$ expression correlated only with tumour size. We conclude that the expression of p21$^{WAF1/CIP1}$ normally depends on active *p53*, but that there may also exist *p53*-independent pathways of induction that reduce the correlation of p21$^{WAF1/CIP1}$ to clinicopathological features.

**Keywords:** pancreatic cancer; pancreatic cancer cell line; p53; WAF1/CIP1

Inactivation of the *p53* tumour-suppressor gene is a frequent genetic abnormality in human cancers (Hollstein et al, 1991). The wild-type *p53* gene encodes a 53-kDa nuclear phosphoprotein that is involved in the regulation of the cell cycle and apoptosis. Its abnormalities are thought to contribute to tumour development (Kastan et al, 1992). Since the finding that *p53* could bind to DNA in a sequence-specific manner and activate other genes (Kern et al, 1991; Vogelstein et al, 1992), the downstream regulation of the *p53*-suppressing function has been vigorously investigated. p21$^{WAF1/CIP1}$ was originally described as a potent inhibitor of cyclin-dependent kinases (Harper et al, 1993) and at the same time was reported as being a $M_r$ 21 000 protein transcriptionally activated by wild-type *p53* (el Deiry et al, 1993). Subsequent studies supported this *p53*-dependent expression of p21$^{WAF1/CIP1}$ (Kern et al, 1991; el Deiry et al, 1994). However, recent studies have suggested that there is a *p53*-independent pathway to induce the expression of p21$^{WAF1/CIP1}$ in muscle and other terminally differentiating cells (Parker et al, 1995) in pancreatic carcinoma (DiGiuseppe et al, 1995) and in leukaemic cells (Schwaller et al, 1995).

In pancreatic cancer, several investigators have reported a mutation frequency of 33–44% (Casey et al, 1993; Scarpa et al, 1993). To date, with regard to the expression of p21$^{WAF1/CIP1}$ in pancreatic cancer, only a single report has been published that indicates the possibility of a *p53*-independent pathway (DiGiuseppe et al, 1995). In the present study, we investigated the correlation between p53 overexpression and the expression of p21$^{WAF1/CIP1}$ as well as p21$^{WAF1/CIP1}$ messenger RNA (mRNA) levels in pancreatic ductal adenocarcinoma tissues and pancreatic cancer cell lines. Furthermore, we were able to induce WAF1/CIP1 in a cell line containing mutant *p53* under serum starvation or hypoxic conditions. Our results demonstrate a statistically significant correlation between p21$^{WAF1/CIP1}$ and active *p53* in human pancreatic cancer, supporting the hypothesis that the suppressor function of *p53* could be mediated through the induction of WAF1/CIP1 (el Deiry et al, 1993; Harper et al, 1993).

*Received 14 November 1996*
*Revised 20 January 1997*
*Accepted 22 January 1997*

*Correspondence to:* HG Beger, Department of General Surgery, University of Ulm, Steinhövelstr. 9, 89075 Ulm, Germany

### MATERIALS AND METHODS

### Patients and tissues

The group of patients with ductal adenocarcinomas of the pancreas included 57 patients who had undergone a pancreaticoduodenectomy or left resection of the pancreas at the Department of General Surgery, University of Ulm. The mean age of the patients was 61.7 years, with a range from 39 to 86 years. Tissues were collected immediately after surgical removal, snap-frozen in liquid nitrogen and stored at –80°C, or they were fixed in 4% formalin for 1 day at room temperature, processed and embedded in paraffin.

### Pancreatic cancer cell lines and culture condition

Human pancreatic adenocarcinoma cell lines Capan-1, Capan-2, AsPC-1, BxPC-3 and MIA PaCa-2 were obtained from the

The first two authors contributed equally to this paper.

**Table 1** Correlation between p53 overexpression and p21 protein expression in 57 cases of pancreatic carcinoma ($P < 0.005$)

| | WAF1/CIP1 protein expression | | |
|---|---|---|---|
| **p53 accumulation** | **Positive** | **Negative** | **Total** |
| Negative | 22 | 12 | 34 |
| Positive | 6 | 17 | 23 (40%) |
| Total | 28 (49%) | 29 | 57 |

**Figure 1** Immunohistochemistry of serial sections of pancreatic ductal adenocarcinoma tissue with negative p53 staining. No p53 staining was achieved (**A**) whereas p21$^{WAF1/CIP1}$ was expressed at corresponding sites (**B**)

**Figure 2** Immunohistochemistry of pancreatic ductal adenocarcinoma tissue with p53 overexpression. p53 staining showed a clear nuclear p53 accumulation (**A**) whereas p21$^{WAF1/CIP1}$ was not detectable at corresponding sites (**B**)

American Type Culture Collection (Rockville, MD, USA), and PMH2/89 was established in our laboratory. Cell lines were incubated in RPMI-1640 medium (GIBCO BRL) supplemented with 10% heat-inactivated fetal bovine serum and 0.5% penicillin and streptomycin in a humidified incubator at 37°C in an atmosphere of 5% carbon dioxide and 95% air. Approximately $1 \times 10^6$ cells of each cell line were enzymatically harvested and were subjected to isolation of mRNA. For further investigation, $1 \times 10^6$ BxPC3 cells in each 10-cm Petri dish were cultured with 7 ml of medium for 48 h. Three dishes containing $1 \times 10^6$ cells were incubated under different conditions: in the control sample, medium was changed at 24 h; in the second sample, medium was not changed for 48 h; and in the third one, medium was not changed for 48 h and air was replaced with 100% carbon dioxide gas to displace oxygen and induce hypoxic conditions. After a 48-h incubation, $1 \times 10^6$ cells of each dish were harvested and used for extraction of mRNA or protein samples.

### Immunohistochemistry

Consecutive 5-µm-thick paraffin-embedded sections were placed on 1% silane-coated slide glasses and were boiled up in 0.2% citrate buffer for 20 min. The two specific monoclonal antibodies DO1 and WAF1 (Oncogene Science, MA, USA), which recognize human p53 protein or WAF1/CIP1 protein (p21$^{WAF1/CIP1}$), respectively, were used for immunohistochemical staining. Optimal results for DO1 were obtained at an antibody dilution of 1:500 and for p21$^{WAF1/CIP1}$ at an antibody dilution of 1:25. The primary antibody was detected with a biotinylated anti-mouse IgG secondary antibody and streptavidin–peroxidase complex (Dako, Denmark), followed by incubation with diaminobenzidine tetrahydrochloride as the substrate. The slides were counterstained with Mayer's haemalaun. On the basis of microscopic observation, staining for both p53 and p21$^{WAF1/CIP1}$ were graded as absent, focal or diffuse.

### Enzyme-linked immunosorbent assay (ELISA)

Protein lysates were extracted from 100 mg of frozen pancreatic adenocarcinoma tissues using lysis buffer (50 mM Tris-HCl, 150 mM sodium chloride, 10 mM EDTA, 1% nonylphenyl-polyethylene

 © Cancer Research Campaign 1997

**Figure 3** Quantitative p53 and WAF1/CIP1 determination in pancreatic carcinoma tissues in relation to the immunohistological results. Tissues with a p53 overexpression in immunohistochemistry showed significantly higher p53 levels in ELISA than p53-negative tissues ($P = 0.001$). Similar results were obtained in $p21^{WAF1/CIP1}$ tissue concentrations compared with immunohistology ($P = 0.002$)

**Table 2** Correlation between p53 overexpression and WAF1/CIP1 protein and mRNA expression in 20 cases of pancreatic carcinoma

| | WAF1/CIP1mRNA expression | | | WAF1/CIP1 protein expression | | |
|---|---|---|---|---|---|---|
| **p53 accumulation** | **Positive** | **Negative** | ***P*-value** | **Positive** | **Negative** | ***P*-value** |
| Negative | 13 | 2 | | 7 | 8 | |
| Positive | 1 | 4 | | 0 | 5 | |
| Total | 14 | 6 | $P < 0.005$ | 7 | 13 | $P < 0.05$ |

glycol, 100 mM phenyl methyl sulphonyl fluoride, 100 mM sodium orthovanadate). The protein concentrations were adjusted at 10 mg $ml^{-1}$ with lysis buffer. For detection of p53 and $p21^{WAF1/CIP1}$, sandwich enzyme immunoassays (Pantropic p53 quantitative ELISA and $p21^{WAF1/CIP1}$ ELISA: Oncogene Science, MA, USA) were used, according to the manufacturer's instructions.

### Western blot analysis

For Western blot analysis, $1 \times 10^6$ cells of each sample were washed three times with phosphate-buffered saline (PBS) and protein was extracted as described above. After denaturation at 95°C for 5 min, 100 μg of protein per lane were electrophoresed on a sodium dodecyl sulphate (SDS)-polyacrylamide gel and transferred to nitrocellulose membranes. The membranes were blocked with PBS containing 5% dried milk and 0.1% Tween-20 and then probed with the p53 monoclonal antibody DO-1 (Oncogene Science). After washing in PBS containing 0.1% Tween-20 and subsequent incubation with horseradish peroxidase-linked anti-mouse second antibody, specific complexes were detected using a chemiluminescent technique (ECL, Amersham Life Science).

### Reverse transcription and polymerase chain reaction

Messenger RNA (mRNA) was isolated from 100 mg of frozen tissue or $1 \times 10^6$ cells of trypsinized cell line using a guanidinium thiocyanate method and oligo(dT)-cellulose column chromatography (QuickPrep Micro mRNA Purification Kit, Pharmacia Biotech) and dissolved in the elution buffer provided in the kit in a final volume of 30 μl.

Complementary DNA (cDNA) was prepared by reverse transcription (RT) of mRNA (5 μl) using SuperScript RT RNAase H-Reverse Transcriptase (GIBCO BRL) and diluted with distilled water in a final

© Cancer Research Campaign 1997 

**Figure 4** RT-PCR of WAF1/CIP1 mRNA of pancreatic ductal adenocarcinoma tissues. The five tissues with wild-type *p53* (as confirmed by SSCP, data not shown) exhibited a clearly positive WAF1/CIP1 PCR signal, whereas only one out of five tissues with *p53* mutations (as confirmed by SSCP) exhibited a WAF1/CIP1 PCR signal (OD, organ donor)

**Figure 5** Western blot of p53 and RT-PCR of p21$^{WAF1/CIP1}$ of pancreatic adenocarcinoma cell lines. Wild-type *p53* cell lines showed no detectable *p53* levels in Western blot but did show signals in WAF1/CIP1 RT-PCR, whereas mutant *p53* cell lines showed an overexpression of p53 in Western blot but no signal in WAF1/CIP1 RT-PCR

volume of 50 µl. WAF1/CIP1 sequences were amplified using the following primers: 5′-AGGATCCATGTCAGAACCGGCTGG-3′ and 5′-CAGGATCCTGTGGGCGGATTAGGGCT-3′. A 6-µl aliquot of cDNA was amplified with 0.125 µM of each primer in a reaction mix comprising 4 µl of 10 × PCR buffer, 2.5 mM dNTPs and 5 units of *Taq* polymerase (Perkin Elmer) in a final volume of 40 µl. Following denaturation at 94°C for 3 min, 31 cycles of amplification consisting of denaturation (1 min at 94°C), annealing (1 min at 60°C) and elongation (1 min at 72°C) were followed by a final extension reaction at 72°C for 5 min. The 520-bp PCR products were electrophoresed on 1.25% small DNA agarose gels containing ethidium bromide and visualized under ultraviolet light. β-Actin was used as an internal standard in each experiment to confirm equal loading of the gel, according to a recently published protocol (du-Breuil et al, 1993).

**Figure 6** Western blot of p53 and RT-PCR of WAF1/CIP1 of BxPC3 under conditions of serum starvation and hypoxia. Serum starvation alone (S) as well as hypoxia plus serum starvation (SH) led to an increase of p53 protein and WAF1/CIP1 mRNA compared with control (F, fresh medium)

### Statistical analysis

Results are expressed as mean values [± standard error (s.e.)]. For statistical analysis, chi-square test, Fisher's exact test and unpaired student *t*-test were used. Significance was defined as $P < 0.05$.

### RESULTS

### Immunohistochemistry of pancreatic cancer tissues

Immunohistochemical analysis of pancreatic ductal adenocarcinoma tissues using DO-1 antibody directed against both wild-type p53 and mutant p53 proteins revealed positive nuclear immunoreactivity in 40% (23 of 57). The anti-p21$^{WAF1/CIP1}$ antibody stained 49% (28 of 57) of the tissues (Table 1). Twenty-two of the 34 patients with p53-negative staining (65%) expressed positive nuclear staining for p21$^{WAF1/CIP1}$ (Figure 1). In contrast, of the 23 patients with p53-positive staining, 17 patients (74%) did not show any immunoreactivity for p21$^{WAF1/CIP1}$ (Figure 2). However, positive staining for p21$^{WAF1/CIP1}$ was detectable in six patients (26%) with p53 overexpression. Statistical analysis of these data indicated that the expression of p21$^{WAF1/CIP1}$ correlated significantly with p53 protein overexpression in pancreatic ductal adenocarcinoma tissues ($P < 0.005$).

### ELISA analysis of pancreatic cancer tissues

ELISA analysis for both p53 and p21$^{WAF1/CIP1}$ were performed in 20 pancreatic adenocarcinoma tissues. The mean p53 level in the samples with positive immunoreactivity for DO1 antibody was 1390 ± 630 pg ml$^{-1}$ and in the samples with negative immunoreactivity was 100 ± 80 pg ml$^{-1}$. The mean p21$^{WAF1/CIP1}$ level in the samples with positive immunoreactivity for p21$^{WAF1/CIP1}$ antibody was 4.9 ± 0.8 U ml$^{-1}$, while in the samples with negative immunoreactivity the mean level was 1.9 ± 0.4 U ml$^{-1}$ (Figure 3). A statistical significance was noted between immunohistochemistry and ELISA analysis in both p53 ($P < 0.001$) and p21$^{WAF1/CIP1}$ ($P < 0.002$).

© Cancer Research Campaign 1997

**Table 3** Clinicopathological features of p21 and p53 expression

| | p53 | | | p21 | | |
|---|---|---|---|---|---|---|
| | **Negative** | **Positive** | ***P*-value** | **Negative** | **Positive** | ***P*-value** |
| Total | 34 | 23 | | 29 | 28 | |
| Sex | | | | | | |
| Male | 19 | 10 | | 14 | 15 | |
| Female | 15 | 13 | 0.25 | 15 | 13 | 0.45 |
| Stage (UICC) | | | | | | |
| Stage I and II | 12 | 1 | | 7 | 6 | |
| Stage III | 17 | 20 | I and II vs III and IV | 18 | 19 | I and II vs III and IV |
| Stage IV | 5 | 2 | 0.005 | 4 | 3 | 0.8 |
| Tumour primary | | | | | | |
| T1 | 5 | 0 | | 2 | 3 | |
| T2 | 20 | 8 | T1 and T2 vs T3 | 10 | 18 | T1 and T2 vs T3 |
| T3 | 9 | 15 | 0.004 | 17 | 7 | 0.01 |
| Lymph nodes | | | | | | |
| Negative | 13 | 1 | | 7 | 7 | |
| Positive | 21 | 22 | 0.003 | 22 | 21 | 0.93 |
| Metastases | | | | | | |
| Negative | 29 | 21 | | 25 | 25 | |
| Positive | 5 | 2 | 0.49 | 4 | 3 | 0.72 |
| Grade | | | | | | |
| G1 | 2 | 0 | | 1 | 1 | |
| G2 | 25 | 11 | G1 and G2 vs G3 | 16 | 20 | G1 and G2 vs G3 |
| G3 | 7 | 12 | 0.014 | 12 | 7 | 0.15 |
| Survival (months) | 16 | 10.5 | 0.08 | 12 | 15 | 0.14 |

G, grade (G1, well differentiated; G2, moderately differentiated; G3, undifferentiated).

### RT-PCR analysis of pancreatic cancer tissues

In order to investigate whether there was a correlation between negative p53 immunoreactivity and WAF1/CIP1 mRNA expression, RT-PCR analyses were carried out in 20 pancreatic adenocarcinoma tissues. Of the 15 tissues not overexpressing p53 protein, 13 tissues (87%) exhibited WAF1/CIP1 mRNA signals, including seven tissues with positive $p21^{WAF1/CIP1}$ staining (Table 2). On the other hand, of the five tissues overexpressing p53 protein, only one tissue revealed a positive WAF1/CIP1 mRNA signal and none of these tissues showed $p21^{WAF1/CIP1}$ (Figure 4). Statistical analysis of these data also indicated a significant correlation of WAF1/CIP1 mRNA expression with negative p53 immunoreactivity in pancreatic ductal adenocarcinoma tissues ($P < 0.005$).

### RT-PCR analysis of pancreatic cancer cell lines

Six human pancreatic adenocarcinoma cell lines Capan-2, AsPC-1, PMH2, Capan-1, BxPC-3 and MIA PaCa-2 were used for the investigation of the WAF1/CIP1 mRNA expression. In Capan-1, BxPC-3 and MIA PaCa-2, *p53* mutations in exon 5, exon 6 and exon 7 were confirmed by single-strand conformation polymorphism (SSCP) respectively. In the other cell lines, no *p53* mutation was detectable (data not shown). The three cell lines with wild-type *p53*, i.e. Capan-2, AsPC-1 and PMH2, exhibited clear PCR signals of amplified WAF1/CIP1 cDNA, whereas the cell lines bearing mutant *p53*, i.e. Capan-1, BxPC-3 and MIA PaCa-2, showed only faint or no PCR signals (Figure 5).

### Induction of WAF1/CIP1 in the pancreatic carcinoma cell line BxPC-3

To further investigate the induction of $p21^{WAF1/CIP1}$, we cultured BxPC-3 containing mutant *p53* under three different conditions: with fresh medium (control, F), with medium left for 48 h (S) and with medium left for 48 h in an hypoxic, hypercapnic atmosphere of 100% carbon dioxide (SH). In Western blot analysis for p53, both sample S and sample SH exhibited distinct accumulation of p53 protein compared with the control sample F. RT-PCR analysis also revealed an increasing expression of WAF1/CIP1 mRNA in the samples S and SH (Figure 6).

### Clinical correlations of p53 and $p21^{WAF1/CIP1}$ expression in pancreatic cancer

Similar to previous reports (Pellegata et al, 1994; Gansauge et al, 1996), p53 overexpression was significantly more frequent in advanced stages (UICC stage III and IV), tumours that had already infiltrated adjacent structures (T3) and had metastasized in regional lymph nodes (N1). p53 overexpression was also significantly more often observed in undifferentiated tumours (G3) (Table 3). These correlations were also reflected in terms of patients' survival (p53 positive, 10.5 months; p53 negative, 16 months). Although p53 overexpression correlated significantly with absent expression of $p21^{WAF1/CIP1}$ ($P < 0.005$), correlations of $p21^{WAF1/CIP1}$ expression to clinicopathological features were reduced compared with p53 overexpression; the only significant

© Cancer Research Campaign 1997 

correlation of p21$^{WAF1/CIP1}$ was found with the T stage. p21$^{WAF1/CIP1}$ was significantly more frequently found in tumours that had not infiltrated adjacent structures (Table 3).

### DISCUSSION

p21$^{WAF1/CIP1}$ seems to be essential in the p53-mediated arrest of the cell cycle in response to DNA damage (Xiong et al, 1993; Dulic et al, 1994; el Deiry et al, 1994). Although the WAF1/CIP1 promoter has a p53 binding site and WAF1/CIP1 transcription is activated by wild-type p53 (el Deiry et al, 1993), WAF1/CIP1 can also be activated by p53-independent factors, as p21$^{WAF1/CIP1}$ is also inducible in p53-null cell (Michieli et al, 1994). A better understanding of cell cycle regulation and apoptotic mechanisms in cancer cells in particular would improve anti-cancer therapy as many tumour cells that lack functional p53 are not able to undergo apoptosis in response to DNA-damaging drugs or radiation (Fisher, 1994). In pancreatic cancer, several groups have examined the status of p53, either by sequencing the gene or by immunohistochemical assessment of accumulation of the p53 protein (Barton et al, 1991; Casey et al, 1993; Lee et al, 1993; Scarpa et al, 1993; Pellegata et al, 1994; Redston et al, 1994; DiGiuseppe et al, 1995; Gansauge et al, 1996).

Immunohistochemical detection of p53 is based on the evidence that the p53 protein in normal adult tissues and in cancer tissues with the wild-type *p53* gene has a half-life of about 20 min (Finlay et al, 1988), whereas mutant *p53* can be stable for hours. As immunohistochemistry alone is only an approximation to the real mutation rate, it may underestimate the mutation frequency of the *p53* gene, especially with regard to deletions or non-sense mutations in the *p53* gene that do not lead to p53 immunoreactivity (Borresen et al, 1991). In some cases this could probably explain why some tissues that seem to be wild type in immunohistochemistry do not correspond to the expected p21$^{WAF1/CIP1}$ expression. On the other hand recent reports have shown that the accumulation of p53 is not always due to mutations in the gene (Bourdon et al, 1995), implying that stabilization of the p53 protein can also lead to an overexpression of p53 as shown by others (Wynford-Thomas, 1992). However, many comparative studies have shown that p53 overexpression is a good approximation to the real mutation rate (Hall et al, 1991; Melhem et al, 1995; Nishio et al, 1996) with approximately 90% concordance between immunostaining and gene analysis (Nishio et al, 1996) and a specificity of immunohistochemistry of 91% (Melhem et al, 1995). In addition, the immunohistochemical evidence of the p21$^{WAF1/CIP1}$ expression could depend on the mutational status of the WAF1/CIP1 protein itself. However, WAF1/CIP1 alterations seem to be rare in human malignancies (Shiohara et al, 1994).

In the present series of surgically resected pancreatic adenocarcinoma tissues, p53 negativity in immunohistochemistry correlated not only with p21$^{WAF1/CIP1}$ expression ($P < 0.005$) but also with WAF1/CIP1 mRNA expression ($P < 0.005$), which supports the hypothesis that wt p53 transactivates the WAF1/CIP1 gene (el Deiry et al, 1993; Dulic et al, 1994). However, in 11% (6/57) of the cases investigated, p21$^{WAF1/CIP1}$ protein was immunohistochemically detectable despite a concomitant p53 accumulation, pointing to existing *p53* mutations or at least to *p53* inactivation; either p21$^{WAF1/CIP1}$ expression was induced in a *p53*-independent pathway (Michieli et al, 1994; Akashi et al, 1995) or the underlying *p53* mutations in some cases still permitted the transactivation of the WAF1/CIP1 gene.

In recent approaches it has been shown that not all mutations of *p53* correlate with a loss of transactivation activity (Ory et al, 1994). In general it has been assumed that mutated p53 proteins exert a dominant negative effect on wt *p53* functions. Forrester and colleagues (1995) recently reported that these effects could be cell-type dependent up to a minimal dominant negative effect of transfectant mutants on co-transfected wt *p53* (Forrester et al, 1995); although it is possible that an up-regulation of wt *p53* leads to the transactivation of the WAF1/CIP1 gene as it is known that p53 levels increase under serum starvation conditions in different cell types. In our cell line experiments, we could demonstrate an up-regulation of p53 under serum starvation conditions accompanied by an increase of WAF1/CIP1 mRNA and p21$^{WAF1/CIP1}$ in the cell line BxPC 3, which is mutated in the *p53* gene (Barton et al, 1991).

The analysis of p53 and p21$^{WAF1/CIP1}$ in relation to clinicopathological features revealed a significant correlation between p53 overexpression and tumour stage as well as tumour size, grading and lymph node metastases, as also shown elsewhere (Pellegata et al, 1994; Gansauge et al, 1996). p53 accumulation seems to correlate with poor prognosis (10.5 vs 16 months), although in our study this relationship did not reach statistical significance. As WAF1/CIP1 is a downstream effector of p53 and is induced in $G_1$ arrest and apoptosis (el Deiry et al, 1994), which mediates growth-regulatory functions of p53, one would expect p21$^{WAF1/CIP1}$ to be a supplementary factor for describing more precisely the effects of p53 than does p53 accumulation concerning clinicopathological features. However, analysis of p21$^{WAF1/CIP1}$ showed only a poor correlation between protein expression and clinical outcome despite the significant correlation between p53 and p21$^{WAF1/CIP1}$ expression, which implies the possibility of bypassing the normally p53-dependent p21$^{WAF1/CIP1}$ induction under some physiological conditions in pancreatic carcinoma. As the p21$^{WAF1/CIP1}$ increase occurring under these appropriate conditions could be temporary, the immunohistochemical expression of p21$^{WAF1/CIP1}$ fails to correlate with clinical data but not in the same manner as p53 overexpression, which is mainly because of persistent genetic changes.

### ACKNOWLEDGEMENT

This work was supported by grant no. GA 541/1-1 to Susanne Gansauge from the Deutsche Forschungsgemeinschaft.

### REFERENCES

Akashi M, Hachiya M, Osawa Y, Spirin K, Suzuki G and Koeffler HP (1995) Irradiation induces WAF 1 expression through a p53-independent pathway in KG-1 cells. *J Biol Chem* **270**: 19181–19187

Barton CM, Staddon SL, Hughes CM, Hall PA, O'Sullivan C, Kloppel G, Theis B, Russell RCG, Neoptolemos J, Williamsson RCN, Lane DP and Lemoine NR (1991) Abnormalities of the p53 tumor suppressor gene in human pancreatic cancer. *Br J Cancer* **64**: 1076–1082

Borresen AL, Hovig E, Smith Sorensen B, Malkin D, Lystad S, Andersen TI, Nesland JM, Isselbacher KJ and Friend SH (1991) Constant denaturant gel electrophoresis as a rapid screening technique for p53 mutations. *Proc Natl Acad Sci USA* **88**: 8405–8409

Bourdon JC, D'Errico A, Paterlini P, Grigioni W, May E and Debuire B (1995) p53 protein accumulation in European hepatocellular carcinoma is not always dependent on p53 gene mutation. *Gastroenterology* **108**: 1176–1182

Casey G, Yamanaka Y, Friess H, Kobrin MS, Lopez ME, Buchler M, Beger HG and Korc M (1993) p53 mutations are common in pancreatic cancer and are absent in chronic pancreatitis. *Cancer Lett* **69**: 151–160

DiGiuseppe JA, Redston MS, Yeo CJ, Kern SE and Hruban RH (1995) p53-independent expression of the cyclin-dependent kinase inhibitor p21 in pancreatic carcinoma. *Am J Pathol* **147**: 884–888

© Cancer Research Campaign 1997

Du-Breuil RM, Patel JM and Mendelow BV (1993) Quantitation of beta-actin-specific mRNA transcripts using xeno-competitive PCR. *PCR Methods Appl* **3**: 57–59

Dulic V, Kaufmann WK, Wilson SJ, Tlsty TD, Lees E, Harper JW, Elledge SJ and Reed SI (1994) p53-dependent inhibition of cyclin-dependent kinase activities in human fibroblasts during radiation-induced G1 arrest. *Cell* **76**: 1013–1023

El Deiry WS, Tokino T, Velculescu VE, Levy DB, Parsons R, Trent JM, Lin D, Mercer WE, Kinzler KW and Vogelstein B (1993) WAF1, a potential mediator of p53 tumour suppression. *Cell* **75**: 817–825

El Deiry WS, Harper JW, O'Connor PM, Velculescu VE, Canman CE, Jackman J, Pietenpol JA, Burrell M, Hill DE, Wang Y, Wilman KG, Mercer WE, Kastan MB, Kohn KW, Elledge SJ, Kinzler KW and Vogelstein B (1994) WAF1/CIP1 is induced in p53-mediated G1 arrest and apoptosis. *Cancer Res* **54**: 1169–1174

Finlay CA, Hinds PW, Tan T-H, Eliyahu D, Oren M and Levine AJ (1988) Activating mutations for transformation by p53 produce a gene product that forms a hsc70-p53 complex with an altered half life. *Mol Cell Biol* **8**: 531–539

Fisher DE (1994) Apoptosis in cancer therapy: crossing the threshold. *Cell* **78**: 539–542

Forrester K, Lupold SE, Ott VL, Chay CH, Band V, Wang XW and Harris CC (1995) Effects of p53 mutants on wild-type p53 mediated transactivation are cell type dependent. *Oncogene* **10**: 2103–2111

Gansauge S, Gansauge F, Negri G, Galle P, Müller J, Poch B, Nüssler AK and Beger HG (1996) The role of anti-p53-autoantibodies in pancreatic disorders. *Int J Pancreatol* **19**: 171–178

Hall PA, Ray A, Lemoine NR, Midgley CA, Krauz T and Lane DP (1991) p53 immunostaining is a marker of malignancy in diagnostic cytopathology. *Lancet* **338**: 513–515

Harper JW, Adami GR, Wei N, Keyomarsi K and Elledge SJ (1993) The p21 Cdk-interacting protein Cip1 is a potent inhibitor of G1 cyclin-dependent kinases. *Cell* **75**: 805–816

Hollstein M, Sidransky D, Vogelstein B and Harris CC (1991) p53 mutations in human cancers. *Science* **253**: 49–53

Kastan MB, Zhan Q, El Deiry WS, Carrier F, Jacks T, Walsh WV, Plunkett BS, Vogelstein B and Fornace AJ (1992) A mammalian cell cycle checkpoint pathway utilizing p53 and GADD45 is defective in ataxia-telangiectasia. *Cell* **71**: 587–597

Kern SE, Kinzler KW, Bruskin A, Jarosz D, Friedman P, Prives C and Vogelstein B (1991) Identification of p53 as a sequence-specific DNA-binding protein. *Science* **252**: 1708–1711

Lee CS, Rush M, Charalambous D and Rode J (1993) Alimentary tract and pancreas: immunhistochemical demonstration of the p53 tumor suppressor gene product in cancer of the pancreas and chronic pancreatitis. *J Gastroenterol Hepatol* **8**: 465–469

Melhem MF, Law JC, El-Ashmawy L, Johnson JT, Landreneau RJ, Srivastava S and Whiteside TL (1995) Assessment of sensitivity and specificity of immunohistochemical staining of p53 in lung and head and neck cancers. *Am J Pathol* **146**: 1170–1177

Michieli P, Chedid M, Lin D, Pierce JH, Mercer WE and Givol D (1994) Induction of WAF1/CIP1 by a p53-independent pathway. *Cancer Res* **54**: 3391–3395

Nishio M, Koshikawa T, Kuroishi T, Suyama M, Uchida K, Takagi Y, Washimi O, Sugiura T, Ariyoshi Y, Takahashi T, Ueda R and Takahashi T (1996) Prognostic significance of abnormal p53 accumulation in primary, resected non-small-cell lung cancers. *J Clin Oncol* **14**: 497–502

Ory K, Legros Y, Auguin C and Soussi T (1994) Analysis of the most representative tumour-derived p53 mutants reveals that changes in protein conformation are not correlated with loss of transactivation or inhibition of cell proliferation. *EMBO J* **13**: 3496–3504

Parker SB, Eichele G, Zhang P, Rawls A, Sands AT, Bradley A, Olson EN, Harper JW and Elledge SJ (1995) p53-independent expression of p21Cip1 in muscle and other terminally differentiating cells. *Science* **267**: 1024–1027

Pellegata NS, Sessa F, Renault B, Bonato M, Leone BE, Solcia E and Ranzani GN (1994) K-ras and p53 gene mutations in pancreatic cancer: ductal and nonductal tumours progress through different genetic lesions. *Cancer Res* **54**: 1556–1560

Redston MS, Caldas C, Seymour AB, Hruban RH, Da Costa L, Yeo CJ and Kern SE (1994) p53 mutations in pancreatic carcinoma and evidence of common involvement of homocopolymer tracts in DNA microdeletions. *Cancer Res* **54**: 3025–3033

Scarpa A, Capelli P, Mukai K, Zamboni G, Oda T, Iacono C and Hirohashi S (1993) Pancreatic adenocarcinomas frequently show p53 gene mutations. *Am J Pathol* **142**: 1534–1543

Schwaller J, Koeffler HP, Niklaus G, Loetscher P, Nagel S, Fey MF and Tobler A (1995) Posttranscriptional stabilization underlies p53-independent induction of p21WAF1/CIP1/SDI1 in differentiating human leukemic cells. *J Clin Invest* **95**: 973–979

Shiohara M, El Deiry WS, Wada M, Nakamaki T, Takeuchi S, Yang R, Chen DL, Vogelstein B and Koeffler HP (1994) Absence of WAF 1 mutations in a variety of human malignancies. *Blood* **84**: 3781–3784

Vogelstein B and Kinzler KW (1992) p53 function and dysfunction. *Cell* **70**: 523–526

Wynford-Thomas D (1992) p53 in tumor pathology: can we trust immunocytochemistry? *J Pathol* **166**: 329–330

Xiong Y, Hannon GJ, Zhang H, Casso D, Kobayashi R and Beach D (1993) p21 is a universal inhibitor of cyclin kinases. *Nature* **366**: 701–704

© Cancer Research Campaign 1997